# Health-related quality of life advantage of long-acting injectable antipsychotic treatment for schizophrenia: a time trade-off study

**DOI:** 10.1186/1477-7525-10-35

**Published:** 2012-04-02

**Authors:** Richard H Osborne, Andrew Dalton, Judy Hertel, Rudolf Schrover, Dell Kingsford Smith

**Affiliations:** 1 Public Health Innovation, Population Health Strategic Research Centre, Deakin University, 221 Burwood Highway, Burwood, VIC, Australia 3125; 2 Centre for Health Policy, Programs and Economics, University of Melbourne, 207 Bouverie Street, Carlton, VIC, Australia 3053; 3 Janssen-Cilag Pty Ltd, 1-5 Khartoum Road, Macquarie Park, New South Wales, Australia 2113; 4 PRIMA Consulting Group Pt Ltd, Suite 1A, Level 2, 802 Pacific Hwy, Gordon, NSW, Australia 2072

**Keywords:** Quality of life, Time-trade-off, Schizophrenia, Treatment interval, Antipsychotic, Long-acting injection

## Abstract

**Background:**

This study was undertaken to estimate utility values for alternative treatment intervals for long acting antipsychotic intramuscular injections for the treatment of schizophrenia.

**Methods:**

Vignettes were developed using the published literature and an iterative consultation process with expert clinicians and patient representative groups. Four vignettes were developed. The first was a vignette of relapsed/untreated schizophrenia. The other three vignettes presented a standardised picture of well-managed schizophrenia with variations in the intervals between injections: once every 2-weeks, 4-weeks and 3-months. A standardised time trade off (TTO) approach was used to obtain utility values for the vignettes. As a societal perspective was sought, a representative sample of individuals from across the community (Sydney, Australia) was recruited. Ninety-eight people completed the TTO interview. The vignettes were presented in random order to prevent possible ordering effects.

**Results:**

A clear pattern of increasing utility was observed with increasing time between injections. Untreated schizophrenia was rated as very poor health-related quality of life with a mean (median) utility of 0.27 (0.20). The treated health states were rated at much higher utilities and were statistically significantly different (*p *< 0.001) from each other: (1) 2-weekly: mean (median) utility = 0.61 (0.65); (2) 4-weekly: mean (median) utility = 0.65 (0.70); (3) 3-monthly: mean (median) utility = 0.70 (0.75).

**Conclusions:**

This study has provided robust data indicating that approximately a 0.05 utility difference exists between treatment options, with the highest utility assigned to 3-monthly injections.

## Background

Schizophrenia is a serious mental illness characterized by symptoms such as hallucinations, delusions, disorganized communication, poor planning, reduced motivation, and blunted affect [1]. The incidence of schizophrenia is around 15.2 per 100,000 persons per year and the lifetime prevalence is about one percent of any population, irrespective of race, gender or social class [2,3]. While this is relatively low, schizophrenia contributes significantly to the global burden of disease due to its typical onset in early adulthood and long-term persistence or fluctuation of symptoms in around two-thirds of individuals [4].

In 1998, it was estimated that the annual mental health care costs per individual with schizophrenia in Australia amounted to $601 million, or $21 600 per individual [5]. When other costs, including lost productivity, were taken into account, the annual societal cost of schizophrenia for the Australian urban population was $1.44 billion, or $51 600 per individual. The relapsing course of schizophrenia in many individuals underlies the significant societal and personal costs of the disorder. While around 80% of people with schizophrenia recover from their first episode of illness, as many as 80% relapse within five years [6].

Antipsychotic medications are the principal treatment for schizophrenia. However, non-compliance with medication is a serious problem, with between 40 and 60% of people with schizophrenia partially or totally non-compliant with oral antipsychotics [7,8]. This is associated with poor treatment outcomes including poor symptom control, loss of insight, decreased functioning, relapse, and hospital admission [7]. Long-acting injections (LAIs) were initially developed in the 1960s in an attempt to guarantee delivery of prescribed medication thereby improving treatment outcomes and reducing the risk of relapse due to non-adherence [9,10].

Data from developed countries suggests that antipsychotic LAIs are used in around 30% of patients with schizophrenia [11]. LAIs are administered by deep intramuscular injection, either in the deltoid or gluteal muscle, depending on the formulation available. In Australia, three atypical LAI antipsychotics have marketing approval, risperidone (Risperdal Consta®), olanzapine pamoate monohydrate (Zyprexa Relprevv®) and paliperidone palmitate (Invega Sustenna®). Risperidone LAI is administered every 2 weeks, olanzapine LAI is administered every 2 or 4 weeks (depending on the dose), and paliperidone palmitate LAI is administered every 4 weeks.

Longer injection intervals are expected to improve health-related quality of life by minimising the frequency of exposure to deep intramuscular injection pain and the psychological distress associated with the general fear of injections. Less frequent injections may also reduce the disruption to the lives of people with schizophrenia and their families and carers due to a reduced requirement in the frequency of travel to outpatient clinics for administration of a LAI.

Literature searches were conducted concurrently in Embase and Medline in May 2010 to locate published utility studies that measured the incremental utility difference between the administration of long-acting injections for the treatment of schizophrenia. This search confirmed the health-related quality of life impact of variations in the injection frequency for LAI treatment for schizophrenia had not been studied. Thus, the aim of this study was to discover whether there was a difference in health-related quality of life (HRQoL) (utility) between shorter and longer intervals for antipsychotic LAIs (that is, once every 2 weeks, 4 weeks or 3 months) for the management of schizophrenia.

## Methods

### Participants and recruitment

A community sample of 124 individuals was invited to participate in a time trade-off (TTO) interview. The participants were registrants of a market research firm and were systematically sampled from four geographic regions across Sydney, Australia. These regions represented a wide range of sociodemographic characteristics, and a balanced sample across socioeconomic status, age, and sex was recruited. In return for their time and travel, respondents received AUS$45 at the end of each interview. The current study was a component of a larger study to understand both the injection pain experience and whether differences in utility between injection intervals exist. The former component covering the injection pain experience in schizophrenia patients receiving risperidone LAI as their standard of care, not reported here, was undertaken to inform the development of vignettes in the latter part [12]. The study received ethical approval from the Eastern Health Research and Ethics Committee and The Alfred Human Research Ethics Committee. For the time trade off interviews specific ethics approval for the study was not required as the registrants were not patients, no interventions were involved, and they had previously consented to occasional surveys. Median interview length was 15 minutes.

### Vignette preparation and presentation

**Figure 1:**
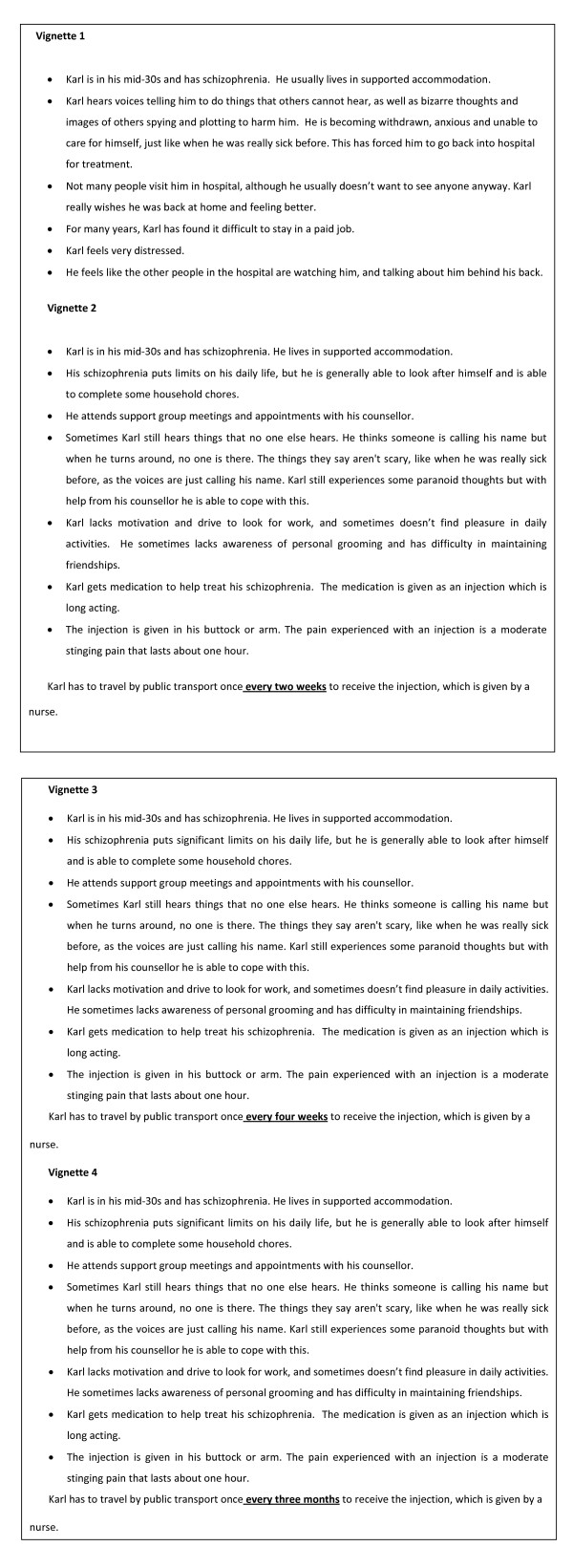
**Vignettes used in time trade off interviews**.

At interview, each participant was presented with standardized background information on schizophrenia and its treatment, together with short descriptions (vignettes or scenarios) of three health states for people with schizophrenia. These vignettes were designed to be clinically and domestically realistic, and easily understood. Each of the vignettes is presented in Figure 1, and can be summarized as follows: (1) a background health state of untreated schizophrenia, (2) treated schizophrenia with injections once every 2 weeks, (3) treated schizophrenia with injections once every 4 weeks, and (4) treated schizophrenia with injections once every 3 months.

An iterative approach was used to develop the vignettes previously utilised by the research team in similar settings [13]. Vignettes were initially drafted by an experienced researcher (RHO) based upon the literature on the quality of life of affected individuals, interrogation of information provided to consumers by a peak consumer organization (SANE; http://www.sane.org), and through published studies on the effectiveness of the interventions. The second iteration resulted from careful review of this initial draft by members of the research team. Draft vignettes were shown to psychiatrists, members of the peak consumer organisation, and people currently treated for schizophrenia to ensure that the descriptions were clinically accurate, reflected the experiences of the target population, and accurately represented the necessary dimensions of HRQoL. This included consultation with people with schizophrenia to derive the terms describing the pain associated with injection. The protocol and specific outcomes for this sub-study will be reported elsewhere [12]. The pain associated with the injection was described in a standardised way--moderate stinging or throbbing pain that lasts about 1 hour--and was derived from data obtained from 57 patients with stable schizophrenia attending an outpatient clinic for injection of an antipsychotic integral to their standard care. Consultation on the content of vignettes continued until consensus was achieved between the researchers, clinicians, and patient representatives. For each set of four vignettes, a female set ("Carol") and a male set ("Karl") were used with participants of the respective gender.

### Time trade-off interviews

Following standard TTO methodology [14,15], participants were asked systematically to make a series of hypothetical trade-offs between living in each of the health states for 10 years, or shorter periods in "normal health." Specifically, participants were asked; "Please consider health state A [one of the test schizophrenia vignettes] and health state B [normal health for 10 years]. Please consider that you have to live in either health state for 10 years and then you die. Which health state would you prefer?" The period of time was varied for the normal health vignette until the point of indifference, where the participant was unable to choose between 10 years in the alternative health state and the period of normal health on offer, the participant implicitly assigned equivalent value to the alternatives offered. The "quality" weight then assigned, known as the level of "utility" in economic evaluation, is the ratio of the time in normal health on offer to the time in the alternative health state (10 years). To avoid ordering bias, respondents were randomized to respond to either health state (2), (3) or (4) immediately after their response to the untreated schizophrenia health state (1). These health states were not labelled in terms of the injection interval included and differed only in their description of the injection interval. The range of possible utility values was a maximum of 1 for full health and a minimum of 0 for death.

Interviewers received training for two full days by an experienced 'master' interviewer. This involved theoretical background, up to eight training interviews with administrative staff, observation of at least 16 interviews and accompanying commentary by the trainer. These initial 16 interviews were undertaken with one interviewer observing to ensure consistency between interviewers. During the study, quality control interviewer meetings were held after every 20-25 interviews. Given that the TTO exercise can be difficult for some interviewees and limited relevant data were available to assist with sample size estimation, 16 initial interviews were conducted. Investigators and interviewers reviewed each initial case to identify potential difficulties participants were having with understanding the TTO technique, whether interviewers were consistent with their oral presentation of vignettes and how they reached the point of indifference, and that the content of the vignettes was acceptable to participants. No anomalies were revealed, so no changes were made to the vignettes or procedures.

### Sample size

Previous work in the area was used to provide initial estimates of the sample size for the study, notably a study of TTO-based utility estimates for different treatment regimes for iron chelation therapy used the same procedures including the same sampling frame [13]. In that study, very large utility differences between treatment regimes were identified (oral vs subcutaneous treatments) with a mean (sd) difference of 0.23 (0.21) in 110 subjects with paired data (beta = 0.8, alpha = 0.05, SD across variables from 0.21 to 0.29, correlation between variables r = 0.67). A sample size estimate indicated that such a study only required about 10 subjects to detect such large differences. Given that the likely difference between the treatment schizophrenia regimes was unknown, but likely to be smaller, a sample size of approximately 100 was specified to enable detection of much smaller incremental differences between health states.

### Statistical procedures

The main statistical exercise was to explore the hypothesis that the utility associated with 3-monthly injections was associated with higher utility than 4-weekly injections and that those 4-weekly injections were associated with higher utility than 2-weekly injections. As the data were paired for individuals, an initial analysis using a two-tailed paired sample *t*-test was used, which was followed by a two-factor analysis of variance with a factor for vignette and a factor for participant to test for overall differences across the vignettes. As the distribution of the data appeared to be not normally distributed, a more conservative overall non-parametric test (Friedman's) was undertaken. Differences in utility values between demographic factors and ordering effects were explored with the Mann-Whitney *U*-test (two groups) or Kruskal-Wallis test (two or more groups). All analyses were undertaken using SPSS version 14 (SPSS Inc., Chicago, IL, USA).

## Results

Of the 124 individuals invited to participate, 98 (78.4%) people were recruited and completed the TTO interview. Fifty per cent were women, 45% reported that they were single (unmarried, divorced or widowed), and about half reported having an income less than AUD$50 000 (see Table 1). Almost half (47%) of participants were under the age of 40 and there was a wide distribution across education groups, with about one quarter having completed year 12 or less, one third having completed vocational education, and the remainder having graduated from university. Just over half were in full time employment and the most common occupational group was business and management (14%), followed by retail and sales (13%). This sample approximates the age and gender mix of Australians where the median age is 36.9 years and there are approximately 13% of people over the age of 65 years [16].

**Table 1:** Health-related quality of life (utility) values for varying treatment intervals across demographic categories; gender, age, income and education

		Vignette 1: Untreated	Vignette 2: 2 weeks	Vignette 3: 4 weeks	Vignette 4: 3 months
**Gender**					
Men	Mean	.22	.57	.62	.70
(N = 49)	Median	.20	.60	.63	.75
	SD	.22	.24	.24	.22
	Mean	.32	.65	.69	.71
Women	Median	.25	.70	.75	.80
(N = 49)	SD	.30	.25	.23	.24
	Z	-1.45	-1.7	-1.6	-.5
	p-value	.15	.09	.11	.6
**Age (years)**					
<30	Mean	.23	.59	.65	.70
(N = 25)	Median	.18	.65	.70	.75
	SD	.23	.22	.22	.26
30-50	Mean	.26	.60	.65	.71
(N = 42)	Median	.15	.63	.70	.75
	SD	.24	.24	.23	.22
>50	Mean	.31	.62	.65	.70
(N = 30)	Median	.20	.70	.73	.79
	SD	.32	.29	.27	.27
	Chi-Square	.52	.91	.10	.13
	df	2	2	2	2
	p-value	.8	.6	.9	.9
**Income (AUD$)**					
<30,000	Mean	.27	.62	.67	.71
(N = 36)	Median	.18	.65	.69	.73
	SD	.30	.24	.24	.22
$30,000- $80,000	Mean	.24	.60	.65	.72
(N = 41)	Median	.18	.65	.70	.80
	SD	.24	.27	.24	.25
>$80,000	Mean	.32	.60	.62	.67
(N = 21)	Median	.40	.65	.70	.70
	SD	.24	.23	.23	.22
	Chi-Square	1.7	.16	.70	1.6
	df	2	2	2	2
	p-value	.4	.9	.7	.4
**Education**					
High school or less	Mean	.27	.62	.67	.73
(N = 25)	Median	.20	.68	.70	.80
	SD	.24	.23	.20	.21
TAFE or college	Mean	.27	.61	.67	.68
(N = 30)	Median	.08	.70	.73	.75
	SD	.31	.28	.26	.25
University graduate	Mean	.27	.58	.63	.70
(N = 41)	Median	.20	.60	.70	.75
	SD	.25	.25	.25	.23
	Chi-Square	1.7	.16	.70	1.6
	df	2	2	2	2
	p-value	.4	.9	.7	.4

As part of interview quality control in the current study, each interviewee was observed for evidence that they had either misunderstood the TTO process or provided illogical answers. Notes on substantive difficulties an interviewee experienced were made at the conclusion of the interview. No interviewees exhibited substantial misunderstanding, therefore, all data were included in the analysis.

**Figure 2:**
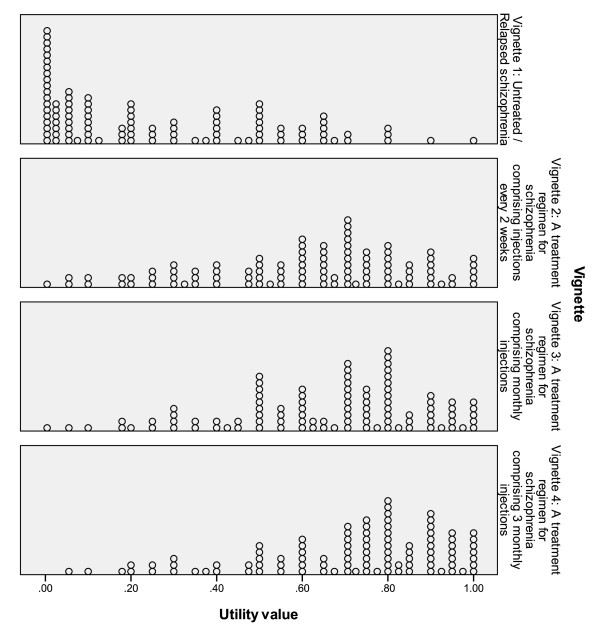
**Frequency distribution of respondents scores across the four treatment regimens (0.0 = death equivalent health-related quality of life, 1.0 = full health)**.

Scores within each vignette were distributed across the entire life/death scale, reflecting widely held beliefs or reactions to trading years of life in the TTO exercise, including the position of a minority of people who refused to trade. The few individuals who refused to trade or traded very little (i.e. with scores >0.80, n = 4) can be seen in Figure 2, Vignette 1 (untreated schizophrenia), at the extreme right of the dot plot. Given that only a few people did not trade, data from all individuals was included in the analysis.

Untreated schizophrenia was rated as the poorest health-related quality of life. The treated health states were all rated at much higher utility and there were clear monotonic differences between the treatment vignettes (see Table 2). Differences between the test vignettes were all statistically significant for both ANOVA and Friedman's test (*p *< 0.001 for all comparisons) (See Table 2). When compared with 2-weekly injections, 4-weekly injections were associated with higher utility (mean [95% CI] 0.047 [0.069-0.025]) and 2-weekly injections were associated with an even larger utility difference compared with 3-monthly injections (0.098 [0.129-0.067]). The difference between 4-weekly injections and 3-monthly injections was also substantial (−0.051 [0.094-0.032]).

**Table 2:** Comparison of health-related quality of life (utility) between vignettes of 2 week, 4 week and 3 month treatment regimens

Vignette	Mean	Median	SD	Min	Max	ANOVA	Friedman Test
Vignette 2 (2 weeks)	0.61	0.65	0.25	0.00	1.00	p<0.001	p<0.001
Vignette 3 (4 weeks)	0.65	0.70	0.24	0.00	1.00		
Vignette 4 (3 monthly)	0.70	0.75	0.23	0.05	1.00		

No systematic differences in utility scores were observed when the order of vignette presentation was considered. Kruskal-Wallis analyses were undertaken across vignette presentation order and no patterns of bias were observed (Untreated schizophrenia Chi sq = 2.6, *p *= 0.8; 2-weekly injections, Chi sq =0.6, *p *= 0.7; 4-weekly injections, Chi sq = 0.9 *p *= 0.1; and 3-monthly injections, Chi sq = 1.3 *p *= 0.9).

## Discussion

This study sought to estimate the strength of preference individuals place on receiving antipsychotic LAIs for schizophrenia every two weeks, every four weeks or every three months. People from the general population were selected to obtain a societal perspective rather than a patient-specific perspective. These community-based preferences reflect utility values as applied in economic evaluations and are more informative than patient-based preferences when making judgments about the allocation of societal resources within health care [14,15]. This is the first study to assess the utility values associated with varying injection frequencies of long-acting antipsychotic treatment administered to patients with schizophrenia.

Our results show that community respondents strongly preferred the health states in which patients receive antipsychotic LAIs less frequently, with a clear pattern of increasing utility observed with increasing time between injections. Administration every four weeks was preferred to administration every two weeks. The mean utility value associated with the health state for 4-weekly injections was 0.65 (median 0.70), indicating that respondents would be prepared to give up 3.5 years of life out of 10 to live in perfect health. In contrast, having schizophrenia that is well-controlled with 2-weekly injections is regarded as a less optimal health state with participants willing to give up 4 years out of 10 to live in normal health. The mean difference between administration for both every two weeks and every three months was 0.1 (median 0.1), equivalent to a 10% difference in scale score. In quality of life research, a change of 10% of a scale is regarded as at least a minimal important difference [17]. The mean utility value associated with the health state in which 3-monthly injections were used was 0.70 (median 0.75), indicating that respondents would be prepared to give up 3 years of life out of 10 to live in perfect health. This implies that most respondents regard having schizophrenia that is well-controlled by 4-weekly or 3-monthly injections as offering a better HRQoL than that controlled by 2-weekly injections.

It is not surprising that participants would prefer less frequent injections given the psychological distress and pain associated with injections, as well as the burden related to the requirement of travel to outpatient clinics for their administration. However, this study provides quantification of the 'value' of this preference; an important consideration in health care funding decisions.

Briggs et al., examined the utility values for relapsed schizophrenia health states in 49 people with stable schizophrenia and in 75 laypersons using a TTO exercise [18]. They found people with schizophrenia were less willing to trade than laypersons to avoid symptoms of schizophrenia (0.60 and 0.45, respectively). The utility of the relapsed state was assessed as less severe than in our study, which may be explained by the severity of our (in hospital) health state, which is substantially worse than their relapse state where the subject is able to work part-time.

Szende et al. [19] used a TTO exercise to investigate preferences for transfusion independence, reduced transfusions and transfusion-dependence in patients with myelodysplastic syndrome. This study's results showed that utility scores for transfusion independence were significantly higher than for reduced transfusions or transfusion-dependence, with a utility difference of 0.17 between reduced transfusions and transfusion dependence. In another recent study, subcutaneous infusion compared with once-daily oral administration of iron chelation therapy in those with iron overload disorders showed a significant impact of the route of drug administration on the utility value that patients associate with treatment [13]. In that study, utility differences of 0.23 were reported, larger than were observed in the present study. That the utility differences are smaller in the current study is to be expected given the less marked differences between treatment options and likely effect on health-related quality of life compared to the other studies. TTO methodology has also been used to estimate the utility associated with three health states (fear of falling, a "good" hip fracture, and a "bad" hip fracture) among older women [20]. A"bad" hip fracture(which results in admission to a nursing home) was valued at 0.05; a "good" hip fracture (maintaining independent living in the community) at 0.31, and fear of falling at 0.67. The results suggest that the loss of ability to live independently in the community was associated with a considerably reduced health-related quality of life. Similarly, the use of antipsychotic LAIs, particularly those administered less frequently may promote adherence with treatment, and therefore independent living in the community in those with schizophrenia.

In this study, we aimed to ensure the utilities extracted by the TTO exercise resulted in consistent and reliable data. In previous studies, acceptable reliability data have been reported for the TTO technique where intraclass correlation coefficient estimates ranged from 0.61 to 0.88 [20-22]. In the present study, relatively wide ranges of preference scores were observed for all LAI administration intervals (Figure 2), but a clear pattern of higher utility was observed with increasing time between injections. As we presented only four TTO exercises to each participant (untreated schizophrenia, 2-weekly, 4-weekly and 3-monthly administration) there was the possibility of ordering effects. To prevent this from confounding the data we included a randomization procedure for the latter three vignettes (vignette one was always presented first).

A potential weakness of the study is the lack of population-based sampling. The sample did include a number of respondents from higher socioeconomic backgrounds. This study incorporated a purposeful sampling in an attempt to obtain a broad societal perspective, rather than what might be regarded as more rigorous population-based sampling approach. Although the sample did include a substantial number of people from higher socioeconomic backgrounds, no differences in utility values obtained were observed across demographic subgroups. Recent privacy legislation and poor recruitment rates in studies employing population-based sampling designs (as low as 30% in some studies) make it an increasingly problematic sampling technique [23]. The study may also have been improved through the inclusion of cognitive debriefing interviews to ratify respondent's answers and inclusion of formal test-retest studies to assess statistical reliability of the TTO interviews.

For this study we chose to use TTO methodology that complies with standard welfare economic principles that individuals are the best judges of their own welfare. There has been some debate in the literature concerning the relative merits of the theoretical properties of TTO, standard gamble and multi-attribute utility instruments, however empirically they have been shown to produce similar results. Some unresolved controversies remain including uncertainty about participant assumptions concerning their wealth when questions require trade-offs over extended periods. It is also known that the longer the period in question, the less likely respondents are to trade.

## Conclusions

In summary, this study has provided valuable insights into what people regard as the potential advantage of LAIs. These results indicate that society associates a higher utility with increasing time between injections, with 4-weekly and 3-monthly administration of an antipsychotic LAI representing an advance over 2-weekly administration in the HRQoL of patients with schizophrenia. Approximately a 0.05 utility advantage exists between 2-weekly, 4-weekly and 3-monthly administration options considered, with the highest utility assigned to 3-monthly injections. As new medications that can be administered over 3-monthly intervals are in development, the present results will provide important information to facilitate the economic evaluation of these drugs by isolating the benefit to patients of this change in their regimen.

## Abbreviations

HRQoL: Health-related quality of life; LAI: Long-acting injection; TTO: Time trade off.

## Competing interests

RHO is employed by Deakin University and is an independent consultant for Measured Solutions for Health P/L; AD is an independent health economist who has received consultancy fees from Janssen-Cilag Pty Ltd; JH, and DKS are employees of Janssen-Cilag Pty Ltd, which markets Risperdal Consta (risperidone long-acting injection) and Invega Sustenna (paliperidone palimitate long-acting injection), RS is the director of PRIMA Consulting Group Pty Ltd, but was employed by Janssen-.Cilag Pty Ltd at the time the study was conducted.

## Authors' contributions

RHO led the design of the study, undertook the statistical analyses, led the manuscript writing, and approved the final manuscript for submission. AD, JH, RS and DKS assisted with the design and interpretation of the study, revised the manuscript and provided approval of the final manuscript for submission.
